# Synthesis and *in Vitro* Antimicrobial Evaluation of New N-Heterocyclic Diquaternary Pyridinium Compounds

**DOI:** 10.3390/molecules190811572

**Published:** 2014-08-05

**Authors:** Bianca Furdui, Georgiana Parfene, Ioana Otilia Ghinea, Rodica Mihaela Dinica, Gabriela Bahrim, Martine Demeunynck

**Affiliations:** 1 Department of Chemistry, Physics and Environment, Faculty of Science and Environment, “Dunarea de Jos” University of Galati, 111 Domneasca Street, 800201 Galati, Romania; E-Mail: bfurdui@ugal.ro; 2 Department of Food Science, Food Engineering and Applied Biotechnology, Faculty of Food Science and Engineering, “Dunarea de Jos” University of Galati, 111 Domneasca Street, 800201 Galati, Romania; E-Mails: Georgiana.parfene@ugal.ro (G.P.); ioana.ghinea@ugal.ro (I.O.G.); bahrim.gabriela (G.B.); 3 Centre National de la Recherche Scientifique (CNRS), UMR 5063, Department of Molecular Pharmacochemistry, F-38041 Grenoble, France; E-Mail: martine.demeunynck@ujf-grenoble.fr; 4 Department of Molecular Pharmacochemistry, University of Grenoble-Alpes, F-38041 Grenoble, France

**Keywords:** pyridinium quaternary salts, antimicrobial activity, 4-[2-(pyridin-4-yl)ethyl]pyridine, 4,4'-bipyridine

## Abstract

A series of bis-pyridinium quaternary ammonium salts (bis-PyQAs) with different aryl and heteroaryl moieties were synthesized and their antimicrobial activity investigated. The inhibition effect of the compounds was evaluated against bacteria, molds and yeasts; the activities were expressed as the minimum inhibitory concentrations (MIC). The relationships between the structure descriptors (logP, polarizability, polar surface area (2D), van der Waals area (3D)) and the biological activity of the tested bis-PyQAs are discussed.

## 1. Introduction

Quaternary ammonium salts and their variously substituted derivatives are important biological agents and have found applications in different fields [1,2,3,4]. In particular, they are used as excellent antimicrobial agents against both Gram-positive and Gram-negative bacteria [5,6,7,8,9,10]. Some of these compounds have antifungal, hemolytic and cytotoxic properties [11]. Quaternary ammonium salts are also widely used for paint, water treatment, textile and sanitizing food preparation areas, because of their relatively low toxicity and broad antimicrobial activity [12,13]. Pyridinium salts constitute a versatile class of compounds used as phase transfer catalysts [14], photosensitizing dyes in polymerization reactions [15] and cationic surfactants [16]. Moreover, pyridinium species have played a crucial role in the development of cycloaddition reactions in heterocycle synthesis and have been extensively used in organic synthesis [17]. Selected salts are also found as ionic liquids [18], and viologens (4,4'-bipyridinium salts) demonstrate electrochromic properties [19].

In recent years, a significant part of the research in heterocyclic chemistry has been devoted to the synthesis and study of variously substituted pyridinium salts. The general synthesis of these salts involves the reaction of pyridine derivatives with reactive halides [20,21,22,23]. This paper focuses on the synthesis and characterization of bis-pyridinium quaternary ammonium salts (bis-PyQAs) starting from 4,4'-bipyridine or 4-[2-(pyridin-4-yl)ethyl]pyridine, as well as the evaluation of their antimicrobial activity. The antimicrobial properties were evaluated using different spoilage microorganisms, which can be found in food microbiota. The relationships between structure, molecular hydrophobicity, solubility, and the biological activity of the tested bis-PyQAs are also discussed.

## 2. Results and Discussion

### 2.1. Synthesis

**Scheme 1:**
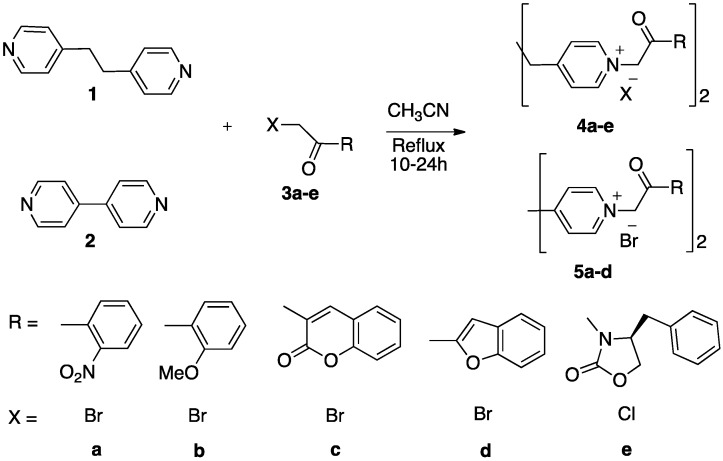
Synthesis of diquaternary salts of bispyridinium.

All nine derivatives were prepared using the methodology shown in Scheme 1. Thus, the synthesis of the diquaternary bispyridinium salts **4a**–**e** has been carried out through the alkylation of 4-[2-(pyridin-4-yl)ethyl]pyridine **1** with reactive halogenated reagents in anhydrous acetonitrile, which seems to be the most convenient method reported in the literature [21]. The diquaternary bispyridinium salts **5a**–**d** were prepared using similar conventional methodology [22], from 4,4'-bipyridine **2**. The products were easily purified by washing with boiling solvent.

**Scheme 2:**
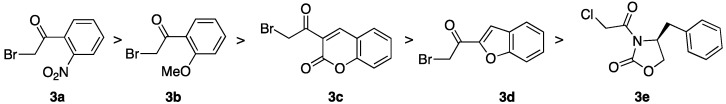
The reactivity of α-halo ketones in nucleophilic substitution.

The alkylation reaction was influenced by the type of substituent present on the alkylating reagent, and it appeared that the reactivity of the -halo ketones followed the order depicted in Scheme 2. The lower reactivity of **3e** is likely due to the lower reactivity of the chloro-alkyl reagent.

The structures of all compounds were confirmed by IR, ^1^H-NMR, COSY, ^13^C-NMR, MS and elemental analysis, whereas their purity was confirmed by HPLC. 

IR spectra of all compounds have been investigated in the frequency range 4,000–650 cm^−^^1^ and the observed absorption bands support the proposed structures: 3,375–3,001 cm^−^^1^ (CH arom), 2,988–2,820 cm^−^^1^ (CH aliph), 1,747–1,667 cm^−^^1^ (C=O), 1,645–1,637 cm^−^^1^ (C=N), 1,525–1,519 and 1,342–1,338 cm^−^^1^ (NO_2_), 1,245–1,241 cm^−^^1^, 1,201–1,188 cm^−^^1^ (C-O-C) and ~996–923 cm^−^^1^ (C-C aliph).

The structures of salts were further confirmed by ^1^H-NMR and COSY analysis. All of these compounds showed multiple peaks in the region of 9.65–7.20 ppm that were assigned to aromatic protons. Additionally, the observed peaks at 6.47–6.14 ppm corresponded to the CH_2_ units linked to the cationic nitrogen, whereas the peaks centered at 3.52–3.47 ppm were attributed to the ethylene bridge protons of Compounds **4a**–**e**. For salt **4e**, each proton of the benzyl moiety, and those from the oxazolidinone ring appeared as coupled multiplets due to its specific configuration. 

^13^C-NMR spectra also confirmed the structures. Thus, for all compounds, the signals of the quaternary carbon of carbonyl groups were found at 192.83–165.74 ppm, and the signals of the lactone carbonyl present in **4c**, **4e** and **5c** were observed at 160.53–158.73 ppm. At higher fields, the signals at 69.98–61.04 ppm represented the CH_2_ groups close to the pyridinium nitrogens, in **5a**–**5e**, whereas the signals at ~33 ppm were attributed to the ethylenic carbons of salts **4a**–**4e**. Due to the ionic structure of the synthesized compounds, their electrospray mass spectra (ESI-MS) gave major peaks corresponding to the organic ions [M^2+^−H^+^], with masses between 453 and 619. 

### 2.2. Antimicrobial Activity

We have previously investigated the antimicrobial properties of some diquaternary ammonium salts and found that they exerted a significant antimicrobial action against bacteria, yeasts and molds [22,24,25]. In the present study, the compounds were tested against nine different microorganisms: Gram-positive bacteria (*Bacillus subtilis*, *Bacillus cereus,* *Sarcina lutea*), yeasts (*Rhodotorula glutinis*, *Candida utilis,* *Saccharomyces cerevisiae*) and molds (*Aspergillus niger*, *Geotrichum candidum, Penicillium roqueforti*). The qualitative results obtained by disk diffusion assay are presented in Table 1.

**Table 1:** Diameters expressed in mm of growth inhibition zone of the bis-pyridinium quaternary ammonium salts (bis-PyQAs) **4a**–**d** and **5a**–**d**. Values are presented as the mean ± SEM. The diameter of the test paper discs is 19 mm.

MO	4a	4b	4c	4d	5a	5b	5c	5d	H_2_O
***B. subtilis***	35.00 ± 0.57	30.33 ± 0.33	19.16 ± 0.16	40.83 ± 0.44	41.33 ± 0.33	45.00 ± 0.57	19.83 ± 0.44	25.33 ± 0.33	0
***B cereus***	41.66 ± 0.33	43.00 ± 0.57	19.33 ± 0.33	37.33 ± 0.33	35.50 ± 0.28	50.50 ± 0.28	19.50 ± 0.28	21.16 ± 0.16	0
***S. lutea***	22.33 ± 0.33	19.50 ± 0.28	41.00 ± 0.57	19.50 ± 0.28	46.00 ± 0.57	45.33 ± 0.33	21.50 ± 0.28	41.50 ± 0.28	0
***S. cerevisiae***	22.00 ± 0.57	22.66 ± 0.66	19.66 ± 0.66	19.16 ± 0.16	19.50 ± 0.28	23.16 ± 0.16	19.33 ± 0.16	19.33 ± 0.33	0
***C. utilis***	22.66 ± 0.66	22.50 ± 0.28	19.33 ± 0.33	19.33 ± 0.33	19.16 ± 0.16	22.66 ± 0.66	19.33 ± 0.33	19.16 ± 0.16	0
***R. glutinis***	40.33 ± 0.33	35.33 ± 0.33	19.50 ± 0.28	19.83 ± 0.44	19.66 ± 0.33	53.50 ± 0.28	19.50 ± 0.28	19.50 ± 0.50	0
***A. niger***	19.66 ± 0.66	19.33 ± 0.33	19.83 ± 0.44	19.33 ± 0.33	19.33 ± 0.33	32.33 ± 0.33	19.33 ± 0.33	19.16 ± 0.16	0
***P. roqueforti***	19.50 ± 0.28	19.16 ± 0.16	19.16 ± 0.16	19.16 ± 0.16	19.16 ± 0.16	19.16 ± 0.16	19.16 ± 0.16	19.50 ± 0.28	0
***G. candidum***	20.00 ± 0.57	21.66 ± 0.66	19.33 ± 0.33	19.33 ± 0.16	19.33 ± 0.33	33.16 ± 0.16	19.16 ± 0.16	19.33 ± 0.33	0

In this test, a compound was considered as active if the difference between the growth inhibition zones of this product and the blank was at least 2 mm. The following classification of the chemical compounds activity was proposed, depending on the dimensions of the inhibition zones (Diz): with low inhibitory effect, Diz ≤ 20 mm; with medium inhibitory effect, Diz = 20–50 mm; and with strong inhibitory effect, Diz ≥ 50 mm [26]. Previous studies showed that the antimicrobial activity of pyridinium quaternary salts is based on their action on the cell wall, and the factors that control the antimicrobial activity include the molecular hydrophobicity [27], adsorbability or electron density of the nitrogen atom [28,29]. When used in water treatment, the high solubility of the antimicrobial agent is mandatory. Indeed, due to the presence of two positive charges on the structures, all compounds displayed good solubility in water. In an effort to establish structure-activity relationships, several parameters were determined. The relative lipophilicity (R_M_) of the tested molecules was measured using thin-layer partition chromatography. Higher R_M_ values indicate greater lipophilicity. Other parameters, including 2D descriptors representing physical properties (logP), 3D descriptors (van der Waals surface area) and quantum chemical parameters (polarizability) were calculated using available on-line chemical software [30]. The data are collected in Table 2. Concerning hydrophobicity, **4b** and **5b** displayed the highest molecular hydrophobicity with R_M_ values of 2.32 and 2.14. The logP parameter was in agreement with R_M_, as exemplified by the low value calculated for 5b. It is worth noting that the bispyridinium derivatives **5a**–**d** showed lower logP and van der Waals surface area values than their analogues containing the ethyl linkage between the two pyridinium rings (**4a**–**d**). The variation of the polar surface area appeared strongly linked to the nature of the R group (see Scheme 1).

**Table 2:** Physicochemical properties of synthesized salts. LogP, polar and van der Waals surface areas and polarizability were calculated using MarvingSketch 6.0.2. R_M_ values are presented as the mean ± SEM.

Compound	mp (°C)	LogP	Polar surface area (Å^2^)	van der Waals surface area (Å^2^)	Polarizability	Hydrophobicity (R_M_)
**4a**	>250	−3.4	133.5	711.4	52.5	2.091 ± 0.027
**4b**	241–243	−3.6	60.3	730.7	53.6	2.325 ± 0.014
**4c**	>300	−3.0	94.5	732.4	59.5	2.122 ± 0.047
**4d**	>300	−3.2	68.2	707.4	57.6	1.938 ± 0.023
**5a**	258–259	−4.3	133.5	652.1	50.3	1.739 ± 0.035
**5b**	252–253	−4.5	60.3	667.1	51.6	2.142 ± 0.024
**5c**	>300	−3.9	94.5	671.0	57.3	1.917 ± 0.049
**5d**	301–302	−4.1	68.2	646.0	55.4	2.092 ± 0.023

If we analyze these data, several general comments can be made. Significantly, all of the salts showed antimicrobial activity with a medium or strong inhibitory effect, against at least one of the tested organisms. Qualitative results revealed that the tested compounds were more effective against bacteria than against yeasts or molds. The three bacterial strains exhibited sensitivity to several molecules, **5a** and **5b** being active against these strains. The most sensitive bacterial strain was *Bacillus cereus*, its growth being strongly or moderately inhibited by six of the tested salts. However, Compounds **4a**–**c** and **5d** proved more active on one strain (*Bacillus cereus* for **4a** and **4b** and *Sarcina lutea* for **4c** and **5d**). If we now tentatively correlate structure and activity, it appears that the most active salts, **5a** and **5b**, present low logP (−4.3 and −4.5, respectively), polarizability (50.3 and 51.6, respectively) and van der Waals surface area (652.1 and 667.1 Å^2^, respectively) compared to the other compounds. However, these two salts differ in polar surface area (133.5 *vs.* 60.3 Ǻ^2^) and R_M_ (1.73 *vs.* 2.14) values. For comparison, the least active salt **5c** has higher values of logP (−3.9), and polarizability (57.3). 

Considering the effect on yeasts, *Rhodotorula glutinis* seemed to be the most sensitive strain, three of the tested chemical compounds (**4a**, **4b** and **5b**) showing strong or moderate activity against it. Again, **5b** emerged as the most active salt. The three salts have high R_M_ values (>2), low logP (−3.4 to −4.5) and low polarizability (51.6-53.6). 

Finally, the bis-PyQAs were marginally active against *Penicillium roqueforti*, and only **5b** salt was moderately active against *Aspergillus niger*. *Geotrichum candidum* was slightly inhibited by only three of the tested chemical compounds. 

To sum up these observations, it is clear that significant differences in the inhibition of the same microorganisms by the different salts were observed. However, it seems possible to distinguish critical structural features. Compounds **5a**–**e** derived from 4,4'-bipyridine are more active than **4a**–**e** derived from 4-[2-pyridin-4-yl)ethyl]pyridine. In each series, the most active molecules (**4a**, **4b** and **5a**, **5b**) bear a phenyl ring as the R substituent. Compound **5b**, containing an electron-donating methoxy group on this phenyl ring, exhibited the highest activity against the Gram-positive bacteria and yeasts. On the other hand, Compounds **4c** and **5c**, containing a chromene ring, showed the lowest activities.

**Figure 1:**
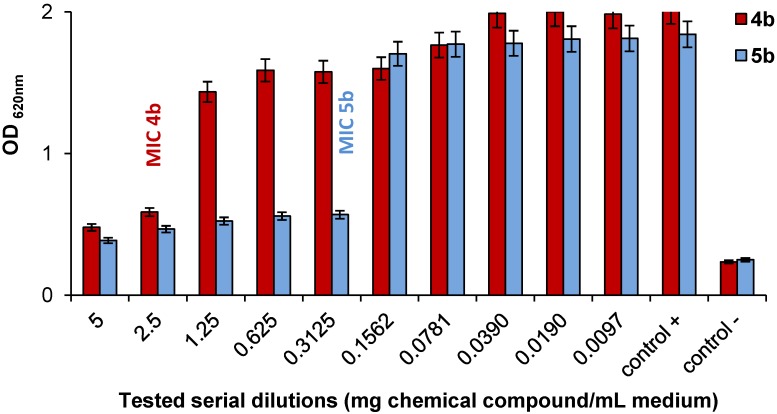
*In vitro* antibacterial activities. Minimal inhibition concentration (MIC) value of **4b** and **5b** against *S. lutea*.

**Figure 2:**
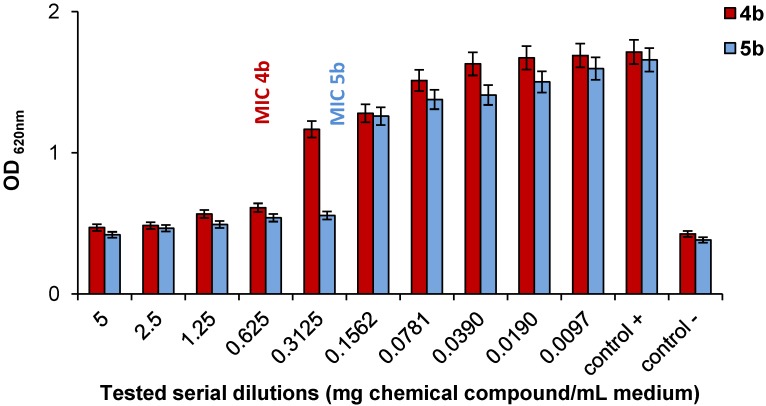
*In vitro* antibacterial activities. Minimal inhibition concentration (MIC) value of **4b** and **5b** against *B. cereus*.

Having these qualitative results in hand, the quantitative evaluation of the antimicrobial efficacy was made by determination of the MIC, for the most active bis-PyQAs, **4b** and **5b**, against two bacterial strains, *Bacillus cereus* and *Sarcina lutea.* The culture growth was evaluated by measuring the increasing turbidity associated with veil formation at the culture surface for the *B. cereus* and with sediment formation for *S. lutea*. From the measured optical densities (OD_620 nm_), it appeared clearly that the highest activity was observed for **5b** with a MIC of 0.3125 mg/mL for both bacterial strains, compared to MIC values of 0.625 mg/mL for *B. cereus* and of 2.5 mg/mL for *S. lutea* for **4b** (Figure 1 and Figure 2).

These data are fully in agreement with the qualitative observations, and Compound **5b** emerges as a promising antibacterial agent. Due to its ionic character, this salt also displays good water solubility despite its rather high hydrophobicity. Our previous studies also showed that these types of antimicrobial agents are heat-resistant [21], which is particularly important if they are incorporated into plastics.

## 3. Experimental Section

### 3.1. General

Reagents used for synthesis were purchased from Aldrich, Fluka and Merck; organic solvents were purchased from Merck. Melting points were recorded with a Stuart SMP10 instrument. IR spectra were recorded from 4,000 to 650 cm^−1^ with a Perkin-Elmer Spectrum 100 instrument by ATR (Attenuated Total Reflectance) technique on a CdSe crystal. ^1^H-NMR, COSY and ^13^C-NMR spectra were recorded with a Bruker 400 Ultrashield spectrometer (at 400 MHz for ^1^H-NMR and at 100 MHz for ^13^C-NMR) operating at room temperature, using DMSO-*d*_6_ as the solvent and TMS as the internal standard. Abbreviations for the data quoted are: s, singlet; d, doublet; t, triplet; q, quartet; dd, doublet of doublets; ddd, doublet of doublet of doublets; m, multiplet. The electrospray ionization (ESI) mass spectra were measured on a Thermo Scientific HPLC/MSQ Plus. Elemental analyses (C, H, N) were performed with a Fisons Instruments 1108 CHNS-O elemental analyzer. The solubility of the compounds was judged by visual observation after agitation in water for 24 h at 25 °C. The evaluation of minimum inhibitory concentration (MIC) was made using a Tecan Infinite 200 PRO NanoQuant Microplate reader. The microplate assay was done in a 96-well microplate, and the absorbance in each well was read at the wavelength of 620 nm.

### 3.2. Synthesis

Experimental details for the synthetic procedures and analytical data and spectra of the pyridinium salts are described. The corresponding bis-pyridine (**1** or **2**) (2 mmol) and halide derivatives (4.4 mmol) were refluxed in acetonitrile (20 mL) for 10 to 24 h. The mixture was cooled to room temperature. The solvent was removed by filtration, and the solid thus obtained was washed with hot acetonitrile. The compounds showed high purity, as assessed by spectroscopic and elemental analyses, and were used without further purification. 

*1-[2-(2-Nitrophenyl)-2-oxoethyl]-4-(2-{1-[2-(2-nitrophenyl)-2-oxoethyl]pyridin-1-ium-4-yl}ethyl)pyridin-1-ium dibromide* (**4a**); Beige crystals; yield 93%; mp > 250 °C (decomposition). IR (cm^−1^): 3,051, 2,895, 2,820, 2,356, 1,715, 1,637, 1,570, 1,519, 1,473, 1,342, 1,220, 1,196, 996. ^1^H-NMR (DMSO-d_6_) δ/ppm: 8.99 (d, *J* = 6.8 Hz, 4H, H_ortho/N+_), 8.34 (d, *J* = 6.8 Hz, 4H, H_meta/N+_), 8.31–8.29 (m, 2H, Ph), 8.12–8.05 (m, 4H, Ph), 7.98–7.94 (m, 2H, Ph), 6.39 (s, 4H, CH_2_/N^+^), 3.52 (s, 4H, CH_2_-CH_2_). ^13^C-NMR (DMSO-*d*_6_) δ ppm: 192.83 (C=O), 161.61 (C), 145.96 (C), 145.45 (CH), 134.68 (CH), 133.25 (C), 131.24 (CH), 128.88 (CH), 127.84 (CH), 124.68 (CH), 66.70 (CH_2_), 33.81 (CH_2_). MS (ESI+), *m/z*: 511 (M^2+^−H^+^). Anal*.* calcd. for C_28_H_24_Br_2_N_4_O_6 _ (M = 672.32 g/mol): C, 50.02; H, 3.60; N, 8.33. Found. C, 49.92; H, 3.71; N, 8.25.

*1-[2-(2-Methoxyphenyl)-2-oxoethyl]-4-(2-{1-[2-(2-methoxyphenyl)-2-oxoethyl]pyridin-1-ium-4-yl}ethyl)pyridin-1-ium dibromide* (**4b**); White crystals; yield 92%; mp 241–243 °C (decomposition). IR (cm^−1^): 3,001, 2,945, 2,876, 2,358, 1,667, 1,639, 1,594, 1,520, 1,484, 1,466, 1,435, 1,337, 1,285, 1,241, 1,207, 1,188, 1,162, 1,112, 1,010, 987. ^1^H-NMR (DMSO-*d*_6_) δ/ppm: 8.95 (d, *J* = 6.8 Hz, 4H, H_ortho/N+_), 8.23 (d, *J* = 6.8 Hz, 4H, H_meta/N+_), 7.90 (dd, *J* = 7.8, 1.6 Hz, 2H, Ph), 7.79–7.74 (m, 2H, Ph), 7.38 (d, *J* = 8.0 Hz, 2H, Ph), 7.19–.15 (m, 2H, Ph), 6.22 (s, 4H, 2CH_2_/N^+^), 4.06 (s, 6H, OCH_3_), 3.48 (s, 4H, CH_2_-CH_2_). ^13^C-NMR (DMSO-*d*_6_) δ ppm: 190.19 (C=O), 160.76 (C), 160.08 (CH), 145.68 (CH), 136.46 (C), 130.40 (CH), 127.24 (CH), 122.86 (C), 120.97 (CH), 113.15 (CH), 69.29 (CH_2_), 56.33 (OCH_3_), 33.74 (CH_2_). MS (ESI+), *m/z*: 481 (M^2+^−H^+^). Anal. calcd. for C_30_H_30_Br_2_N_2_O_4 _(M = 642.38 g/mol): C, 56.09; H, 4.71; N, 4.36. Found. C, 55.99; H, 4.86; N, 4.21.

*1-[2-Oxo-2-(2-oxo-2H-chromen-3-yl)ethyl]-4-(2-{1-[2-oxo-2-(2-oxo-2H-chromen-3-yl)ethyl]pyridin-1-ium-4-yl}ethyl)pyridin-1-ium dibromide* (**4c**); White-beige crystals; yield 90%; mp > 300 °C. IR (cm^−1^): 3,049, 3,022, 2,994, 2,945, 2,875, 2,359, 2,337, 1,698, 1,643, 1,601, 1,556, 1,444, 1,368, 1,344, 1,184, 979. ^1^H-NMR (DMSO-*d*_6_) δ/ppm: 8.96 (s, 2H, chromene), 8.89 (d, *J* = 6.4 Hz, 4H, H_ortho/N+_), 8.21 (d, *J* = 6.4 Hz, 4H, H_meta/N+_), 8.07 (d, *J* = 7.4 Hz, 2H, chromene), 7.85 (t, *J* = 8.4 Hz, 2H, chromene), 7.57 (d, *J* = 8.4 Hz, 2H, chromene), 7.49 (t, *J* = 7.4 Hz, 2H, chromene), 6.27(s, 4H, CH_2_/N^+^), 3.47 (s, 4H, CH_2_-CH_2_). ^13^C-NMR (DMSO-*d*_6_) δ/ppm: 188.09 (C=O), 158.73 (C=O), 154.84 (C), 149.52 (CH), 145.63 (C), 145.50 (CH), 131.39 (C), 128.32 (CH), 127.28 (C), 126.24 (CH), 125.37 (CH), 121.19 (CH), 113.42 (CH), 61.04 (CH_2_), 33.78 (CH_2_). MS (ESI+), *m/z*: 557 (M^2+^−H^+^). Anal*.* calcd. for C_34_H_26_Br_2_N_2_O_6_ (M = 718.38 g/mol): C, 56.84; H, 3.65; N, 3.90. Found: C, 56.99; H, 3.75; N, 3.75.

*1-[2-(1-Benzofuran-2-yl)-2-oxoethyl]-4-(2-{1-[2-(1-benzofuran-2-yl)-2-oxoethyl]pyridin-1-ium-4-yl}ethyl)pyridin-1-ium dibromide* (**4d**); Yellow-beige crystals; yield 76%; mp > 300 °C (decomposition). IR (cm^−1^): 3,010, 2,970, 2,893, 2,631, 1,687, 1,643, 1,612, 1,549, 1,518, 1,474, 1,347, 1,291, 1,155, 1,136, 1,019, 928. ^1^H-NMR (DMSO-*d*_6_) δ/ppm: 9.02 (d, *J* = 6.8 Hz, 4H, H_ortho/N+_), 8.30 (d, *J* = 6.8 Hz, 4H, H_meta/N+_), 8.20 (s, 2H, benzofuran), 7.97 (dd, *J* = 8.0, 1.0 Hz, 2H, benzofuran), 7.85 (dd, *J* = 8.4, 1.0 Hz, 2H, benzofuran), 7.64–7.69 (m, 2H, benzofuran), 7.49–7.46 (m, 2H, benzofuran), 6.43 (s, 4H, CH_2_/N^+^), 3.52 (s, 4H, CH_2_-CH_2_). ^13^C-NMR (DMSO-*d*_6_) δ/ppm: 181.31 (C=O), 161.34 (C), 155.17 (C), 149.36 (C), 145.81 (CH), 129.46 (CH), 127.51 (CH), 126.46 (CH), 125.02 (C), 124.12 (CH), 115.69 (CH), 112.37 (CH), 64.86 (CH_2_), 33.75 (CH_2_). MS (ESI+), *m/z*: 501 (M^2+^−H^+^). Anal*.* calcd. for C_32_H_26_Br_2_N_2_O_4_ (M = 662.37 g/mol): C, 58.03; H, 3.96; N, 4.23. Found: C, 58.18; H, 4.14; N, 4.08.

*1-{2-[(4R)-4-Benzyl-2-oxo-1,3-oxazolidin-3-yl]-2-oxoethyl}-4-[2-(1-{2-[(4S)-4-benzyl-2-oxo-1,3-oxazolidin-3-yl]-2-oxoethyl}pyridin-1-ium-4-yl)ethyl]pyridin-1-ium dichloride* (**4e**); Beige-brown crystals; highly hygroscopic; yield 46%; mp not determined. IR (cm^−1^): 3,375, 3,029, 2,361, 2,337, 1,747, 1,698, 1,639, 1,571, 1,512, 1,518, 1,472, 1,391, 1,276, 1,198, 1,116, 985. ^1^H-NMR (DMSO-*d*_6_) δ/ppm: 9.03 (d, *J* = 6.8 Hz, 4H, H_ortho/N+_), 8.28 (d, *J* = 6.8 Hz, 4H, H_meta/N+_), 7.38–7.27 (m, 10H, Ph), 6.14 (s, 4H, CH_2_/N^+^), 4.72–4.66 (m, 2H, oxazolidine), 4.52–4.48 (m, 2H, oxazolidine), 4.37–4.34 (m, 2H, oxazolidine), 3.49 (s, 4H, CH_2_-CH_2_), 3.13–3.09 (m, 2H, CH_2_/benzyl), 2.98–2.92 (m, 2H, CH_2_/benzyl). ^13^C-NMR (DMSO-*d*_6_) δ/ppm: 165.74 (C=O), 161.40 (COO), 153.42 (C), 145.92 (CH), 135.29 (C), 129.54 (CH), 128.69 (CH), 127.31 (CH), 127.03 (CH), 67.56 (CH_2_), 62.66 (CH_2_), 54.61 (CH), 36.56 (CH_2_), 33.75 (CH_2_). MS (ESI+), *m/z*: 619 (M^2+^−H^+^). Anal*.* calcd. for C_36_H_36_Cl_2_N_4_O_6 _ (M = 691.60 g/mol): C, 62.52; H, 5.25; N, 8.10. Found: C, 62.37; H, 5.55; N, 7.90.

*1-[2-(2-Nitrophenyl)-2-oxoethyl]-4-{1-[2-(2-nitrophenyl)-2-oxoethyl]pyridin-1-ium-4-yl}pyridin-1-ium dibromide* (**5a**); Red crystals; yield 96%; mp 258–259 °C (decomposition). IR (cm^−1^): 3,046, 2,830, 2,773, 1,716, 1,639, 1,525, 1,505, 1,449, 1,360, 1,338, 1,223, 1,203, 997. ^1^H-NMR (DMSO-*d*_6_) δ/ ppm: 9.65 (d, *J* = 8.4 Hz, 4H, H_ortho/N+_), 9.32 (d, *J* = 8.4 Hz, 4H, H_meta/N+_), 8.31 (d, *J* = 7.6 Hz, 2H, Ph), 8.12–8.07 (m, 4H, Ph), 7.98–7.94 (m, 2H, Ph), 6.47 (s, 4H, CH_2_/N^+^). ^13^C-NMR (DMSO-*d*_6_)δ ppm: 191.83 (C=O), 158.75 (C), 157.59 (CH), 146.95 (C), 146.09 (CH), 134.55 (CH), 130.92 (C), 128.92 (CH), 127.00 (CH), 124.63 (CH), 65.98 (CH_2_). MS (ESI+), *m/z*: 483 (M^2+^−H^+^). Anal*.* calcd. for C_26_H_20_Br_2_N_4_O_6_ (M = 644.27 g/mol): C, 48.47; H, 3.13; N, 8.70. Found. C, 48.29; H, 3.28; N, 8.56.

*1-[2-(2-Methoxyphenyl)-2-oxoethyl]-4-{1-[2-(2- methoxyphenyl)-2-oxoethyl]pyridin-1-ium-4-yl}pyridin-1-ium dibromide* (**5b**); Yellow crystals; yield 94%; mp 252–253 °C (decomposition). IR (cm^−1^): 3,036, 2,988, 1,677, 1,641, 1,596, 1,485, 1,460, 1,434, 1,329, 1,280, 1,245, 1,197, 1,111, 1021, 987. ^1^H-NMR (DMSO-*d*_6_) δ/ppm: 9.35 (d, *J* = 7.2 Hz, 4H, H_ortho/N+_), 8.96 (d, *J* = 7.2 Hz, 4H, H_meta/N+_), 7.94 (dd, *J* = 7.9, 1.9 Hz, 2H, Ph), 7.79 (ddd, *J* = 8.5, 7.2, 1.9 Hz, 2H, Ph), 7.41 (dd, *J* = 8.5, 1.0 Hz, 2H, Ph), 7.20 (ddd, 2H, *J* = 7.9, 7.2, 1.0 Hz, 2H, Ph), 6.38 (s, 4H, CH_2_/N^+^), 4.10 (s, 6H, OCH_3_). ^13^C-NMR (DMSO-*d*_6_) δ ppm: 190.89 (C=O), 160.15 (C), 149.22 (CH), 147.21 (CH), 136.59 (C), 130.49 (CH), 126.29 (CH), 122.80 (C), 121.03 (CH), 113.22 (CH), 69.98 (CH_2_), 56.41 (OCH_3_). MS (ESI+), *m/z*: 453 (M^2+^−H^+^). Anal*.* calcd. for C_28_H_26_Br_2_N_2_O_4_ (M = 614.33 g/mol): C, 54.74; H, 4.27; N, 4.56. Found. C, 54.88; H, 4.41; N, 4.37.

*1-[2-Oxo-2-(2-oxo-2H-chromen-3-yl)ethyl]-4-{1-[2-oxo-2-(2-oxo-2H-chromen-3-yl)ethyl]pyridin-1-ium-4-yl}pyridin-1-ium dibromide* (**5c**); Yellow crystals; yield 93%; mp > 300 °C (decomposition). IR (cm^−1^): 3,052, 2,990, 2,931, 1,695, 1645, 1,601, 1,556, 1,506, 1,444, 1,371, 1,213, 1,186, 979. ^1^H-NMR (DMSO-*d*_6_) δ/ppm: 9.27 (d, *J* = 6.6 Hz, 4H, H_ortho/N+_), 9.04 (s, 2H, chromene), 8.93 (d, *J* = 6.6 Hz, 4H, H_meta/N+_), 8.16–8.10 (m, 2H, chromene), 7.96–7.85 (m, 2H, chromene), 7.83 (d, *J* = 8.4 Hz, 2H, chromene), 7.59–7.49 (m, 2H, chromene), 6.44 (s, 4H, CH_2_/N^+^). ^13^C-NMR (DMSO-*d*_6_) δ/ppm: 189.05 (C=O), 160.53 (C=O), 154.14 (C), 149.05 (C), 146.50 (CH), 141.65 (CH), 131.39 (CH), 128.45 (CH), 126.28 (CH), 125.24 (CH), 122.37 (C), 120.19 (C), 115.42 (CH), 63.04 (CH_2_). MS (ESI+), *m/z*: 529 (M^2+^−H^+^). Anal*.* calcd. for C_32_H_22_Br_2_N_2_O_6_ (M = 690.33 g/mol): C, 55.67; H, 3.21; N, 4.06. Found: C, 55.52; H, 3.12; N, 3.89.

*1-[2-(1-Benzofuran-2-yl)-2-oxoethyl]-4-{1-[2-(1-benzofuran-2-yl)-2-oxoethyl]pyridin-1-ium-4-yl}pyridin-1-ium dibromide* (**5d**); Green crystals; yield 78%; mp 301–302 °C (decomposition). IR (cm^−1^): 3,117, 3,055, 2,990, 2,892, 1,684, 1,639, 1,613, 1,550, 1,506, 1,455, 1,357, 1,342, 1,290, 1,155, 1,136, 1,017, 923. ^1^H-NMR (DMSO-*d*_6_) δ/ppm: 9.37 (d, *J* = 7.0 Hz, 4H, H_ortho/N+_), 8.98 (d, *J* = 6.8 Hz, 4H, H_meta/N+_), 8.25 (d, *J* = 1.0 Hz, 2H, benzofuran), 8.01–7.99 (m, 2H, benzofuran), 7.88 (dd, *J* = 8.4, 0.9 Hz, 2H, benzofuran), 7.69 (ddd, *J* = 8.4, 7.2, 1.3 Hz, 2H, benzofuran), 7.50 (ddd, *J* = 8.1, 7.2, 0.9 Hz, 2H, benzofuran), 6.46 (s, 4H, CH_2_/N^+^). ^13^C-NMR (DMSO-*d*_6_) δ/ppm: 181.31 (C=O), 155.23 (C), 150.78 (C), 149.70 (C), 147.34 (CH), 129.58 (CH), 126.61 (CH), 126.46 (CH), 125.25 (C), 124.17 (CH), 115.88 (CH), 112.41 (CH), 65.56 (CH_2_). MS (ESI+), *m/z*: 473 (M^2+^−H^+^). Anal*.* calcd. for C_30_H_22_Br_2_N_2_O_4_ (M = 634.31 g/mol): C, 56.80; H, 3.50; N, 4.42. Found: C, 57.01; H, 3.68; N, 4.32.

### 3.3. Determination of Molecular Hydrophobicity

The chromatographic R_M_ value, related to the logarithm of the partition coefficient, can be used to estimate the molecular hydrophobicity of the bis-PyQAs [24]. The R_M_ values were determined by thin layer partition chromatography, using acetonitrile/ethyl alcohol (10:10) at 25 °C for 30 min. The R_M_ value is defined as R_M_ = log ((1/R_f_) − 1), where R_f_ is the flow rate; R_f_ = (solute velocity/mobile phase velocity).

### 3.4. Antimicrobial Activity

The inhibitory potential of the investigated compounds was tested against nine microorganisms, including Gram-positive bacteria (*Bacillus subtilis*, *Bacillus cereus*, *Sarcina lutea*), yeasts (*Rhodotorula glutinis*, *Candida utilis*, *Saccharomyces cerevisiae*) and molds (*Aspergillus niger*, *Geotrichum candidum*,*Penicillium roqueforti*). All of the bacterial and fungal strains were isolated from food spoilage microbiota and are all included in the collection of microorganisms (coded MIUG) of the Bioaliment Platform, Faculty of Food Science and Engineering, Dunarea de Jos University of Galati. 

The cultures of indicator microorganisms were maintained in medium agar slants at 4 °C and used as stock cultures. The microorganism cultures were prepared from the stock cultures, through cultivation for 24 h at 37 °C on nutrient agar, for bacteria, and for 72 h at 25 °C on Sabouraud agar, for fungi. 

The agar diffusion method was used for the qualitatively assessment of the antibacterial and antifungal properties of the synthesized compounds. Samples (1 mL) of each test microorganism suspension (10^6^ colony-forming units CFU/mL for bacteria and yeasts and of 10^5^–10^6^ spores/mL for molds) obtained from microorganism cultures by serial dilutions were inoculated at 42 °C in sterile agar culture medium, in sterile Petri dishes. After homogenization and solidification of the medium, sterile 19 mm-diameter paper discs, loaded with 30 μL from 5 mg/mL aqueous solutions of each tested bis-PyQA were aseptically placed on the surface. Sterile water-impregnated discs were used as blanks. The plates inoculated with bacteria were incubated at 37 °C for 48 h and those with fungi at 25 °C for 3–5 days. After 24 h of cultivation, the diameters of the inhibition zones (Diz, mm) were measured to the nearest 0.5 mm, compared to the blank, under the same standardized conditions. The evaluation of microbiostatic or microbicide effects of the studied chemical compounds was also made by checking some morphological characteristics of colonies, *i.e.*, pigmentation and sporulation intensity.

The quantitative effect of the substances with the higher inhibitory potential of cells growth was determined *in vitro*, by microbial cultivation in stationary conditions, in liquid nutrient broth medium, on the basis of the minimum inhibitory concentration (MIC) values. The most sensitive microorganisms were used for quantitative evaluation. Serial dilutions (5, 2.5, 1.25, 0.625, 0.3125, 0.1562, 0.0781, 0.0390, 0.0190 and 0.0097 mg of chemical compound/mL medium) were prepared from stock solutions (5 mg/mL); 100 μL of each standardized microbial suspension was added to an equal volume of each chemical compound dilution (excluding the sterility control). After incubation for 24 h ± 1 h at 37 °C for bacteria, the turbidity of the cultures associated with the veil formation at the medium’s surface or sediment formation was visually assessed. The microorganism’s growth was monitored by optical density determination (OD_620 nm_) using the microplate reader. The tests were performed simultaneously on negative controls (only medium), positive controls (medium with test microorganism) and sterility controls (medium with chemical compounds). The lowest concentration of antimicrobial agent that inhibits the development of visible growth was taken as the minimum inhibitory concentration. All of the experiments were performed in triplicate.

## 4. Conclusions

We report here the efficient synthesis of nine new symmetrical diquaternary salts by alkylation of either 4-[2-(pyridin-4-yl)ethyl]pyridine or 4,4'-bipyridine, with various bromo- or chloro-acetophenone analogues. It appeared that the reaction was more efficient with 4,4'-bipyridine compared to 4-[2-(pyridin-4-yl)ethyl]pyridine. The antimicrobial properties for eight salts were investigated through disk diffusion assay, revealing that all compounds display low to high activity against the tested microorganisms and a broad spectrum of activity. Compounds **4a**–**d**, **5a** and **5d** show efficient inhibitory properties at least against one bacterial strain. However, the most active bis-PyQA was Compound **5b**, which showed a broad spectrum of activity. We found some indications that the antibacterial properties seem to correlate well with low logP and van der Waals surface area values.

All of the new compounds showed significant *in vitro* antibacterial activity, the Compounds **4b** and **5b** having superior antimicrobial activity against all microorganisms under investigation. Further studies should be done to elucidate their mechanism of action and to determine whether their activity is lethal, or merely inhibitory to microorganisms. Preliminary studies [31] showed that some of these salts are also able to prevent or destroy biofilm formation with microorganisms from food industry hygiene.

Our results suggest that we have uncovered new leads that may proceed further in the development of antibacterial agents.

## Supplementary Materials

Supplementary materials can be accessed at: http://www.mdpi.com/1420-3049/19/8/11572/s1.

## Supplementary Files

Supplementary File 1
