# Supplementary material for: Synthesis and in Vitro Antimicrobial Evaluation of New N-Heterocyclic Diquaternary Pyridinium Compounds
Source: Molecules. 2014 Aug 5;19(8):11572–85. doi: 10.3390/molecules190811572 (PMC6271789; doi:10.3390/molecules190811572)

Supplementary Information

Figure S1. 1-[2-(2-Nitrophenyl)-2-oxoethyl]-4-(2-{1-[2-(2-nitrophenyl)-2-oxoethyl]pyridin-1-ium-4-yl}ethyl)pyridin-1-ium dibromide (**4a**).

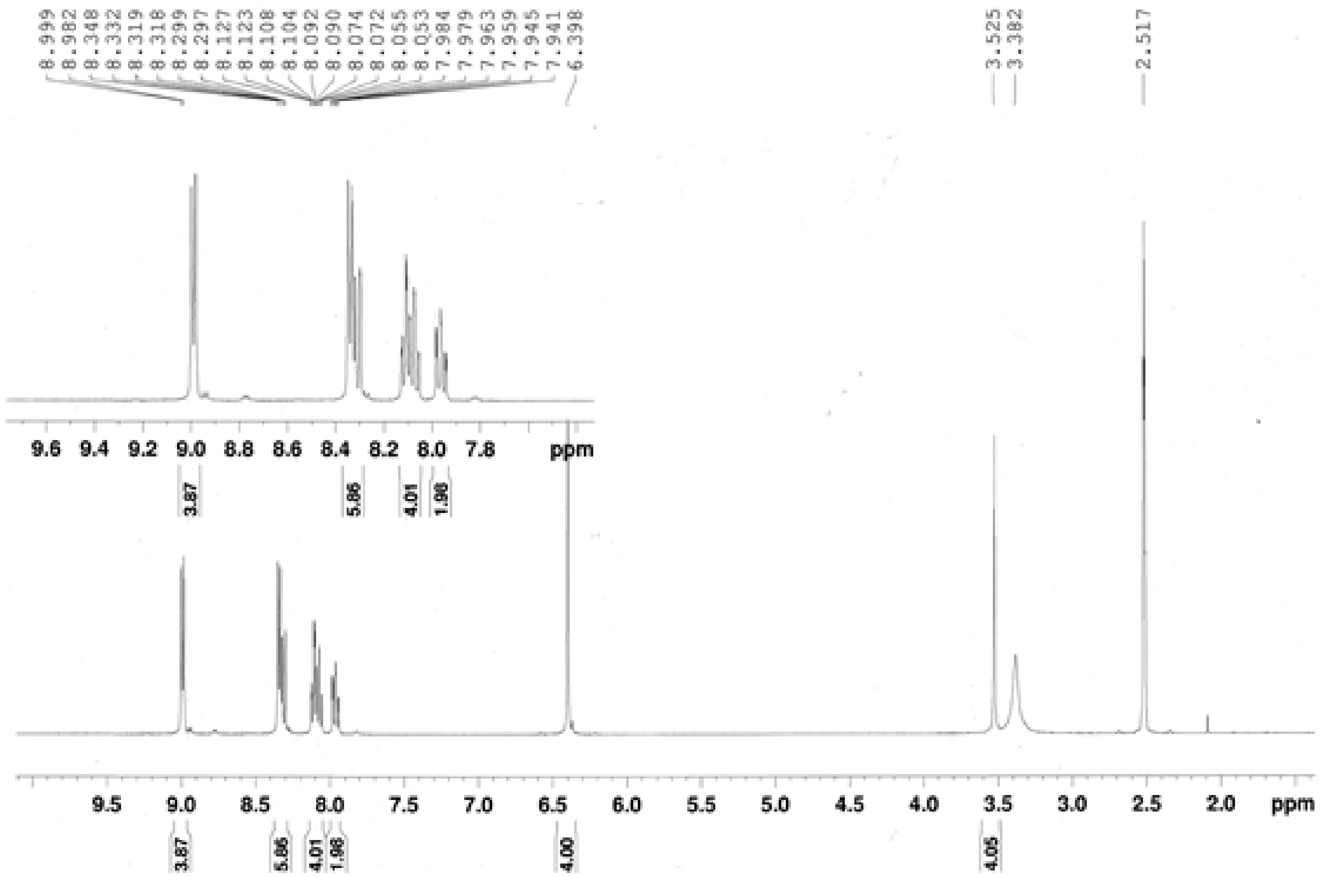

Figure S2. 1-[2-(2-Methoxyphenyl)-2-oxoethyl]-4-(2-{1-[2-(2-methoxyphenyl)-2-oxoethyl]pyridin-1-ium-4-yl}ethyl)pyridin-1-ium dibromide (**4b**).

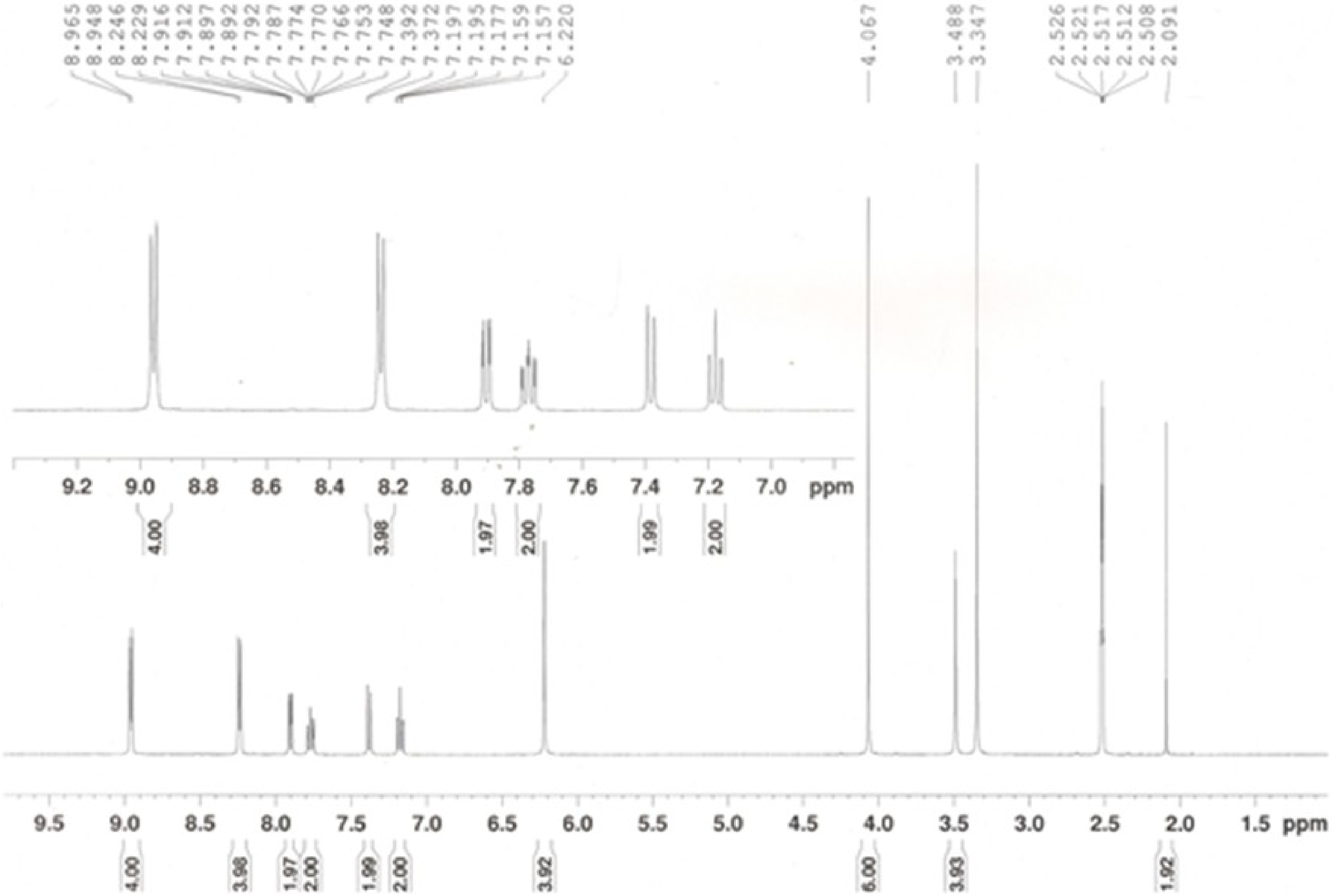

Figure S3. 1-[2-Oxo-2-(2-oxo-2H-chromen-3-yl)ethyl]-4-(2-{1-[2-oxo-2-(2-oxo-2H-chromen-3-yl)ethyl]pyridin-1-ium-4-yl}ethyl)pyridin-1-ium dibromide (**4c**).

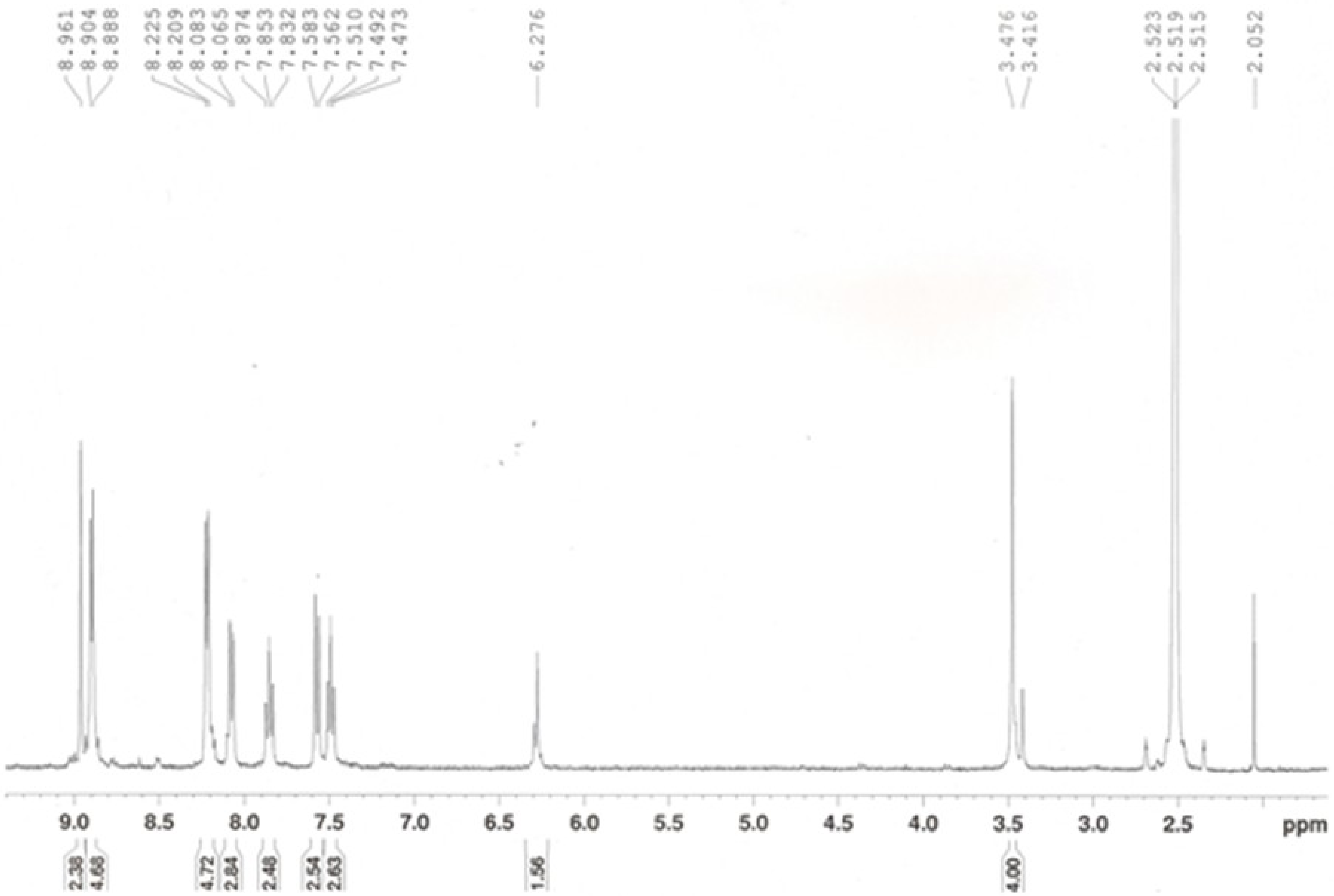

Figure S4. 1-{2-[(4R)-4-Benzyl-2-oxo-1,3-oxazolidin-3-yl]-2-oxoethyl}-4-[2-(1-{2-[(4S)-4-benzyl-2-oxo-1,3-oxazolidin-3-yl]-2-oxoethyl} pyridin-1-ium-4-yl)ethyl]pyridin-1-ium dichloride (**4e**).

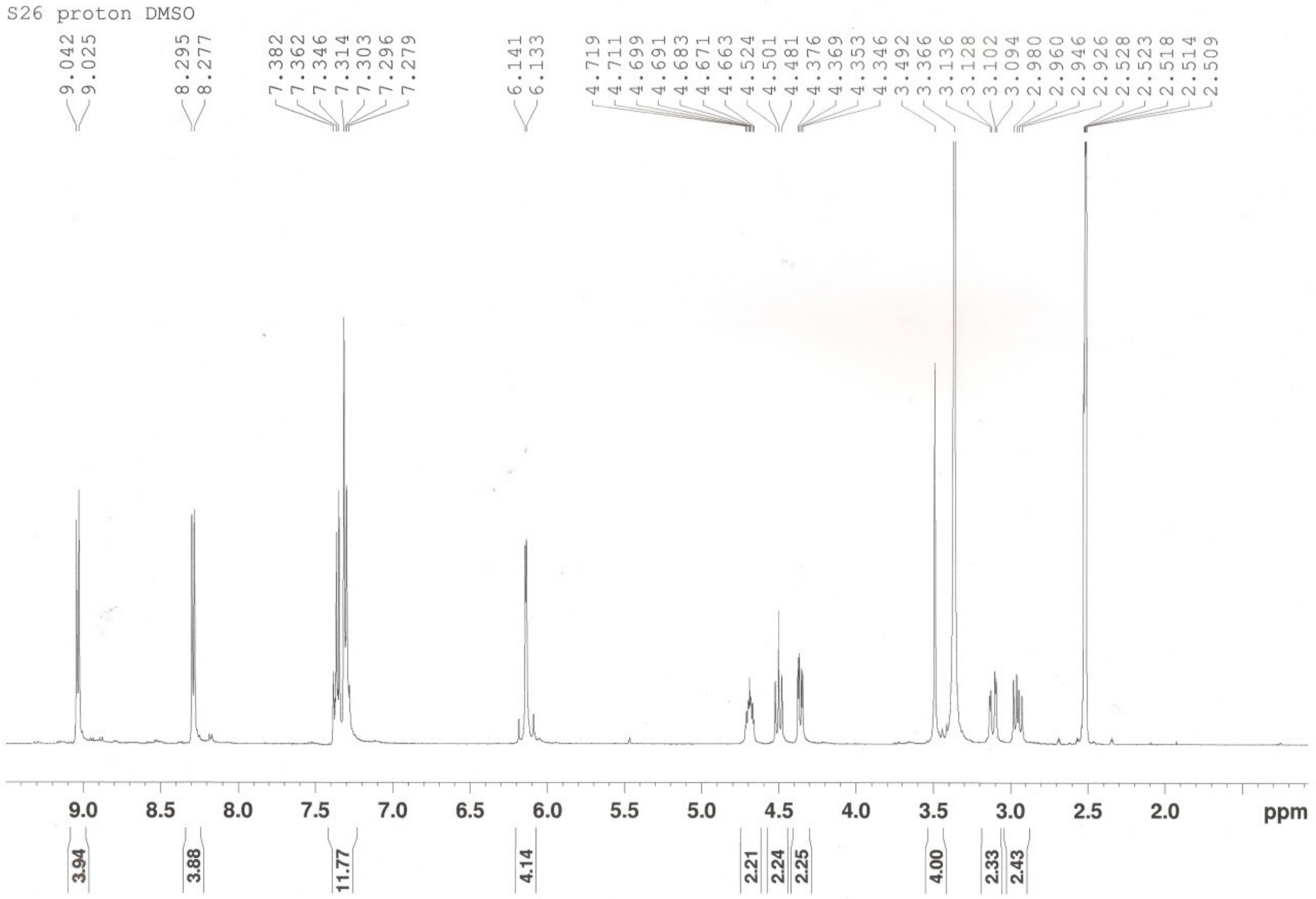

Figure S5. 1-[2-(2-Methoxyphenyl)-2-oxoethyl]-4-{1-[2-(2-methoxyphenyl)-2-oxoethyl]pyridin-1-ium-4-yl} pyridin-1-ium dibromide (**5b**).

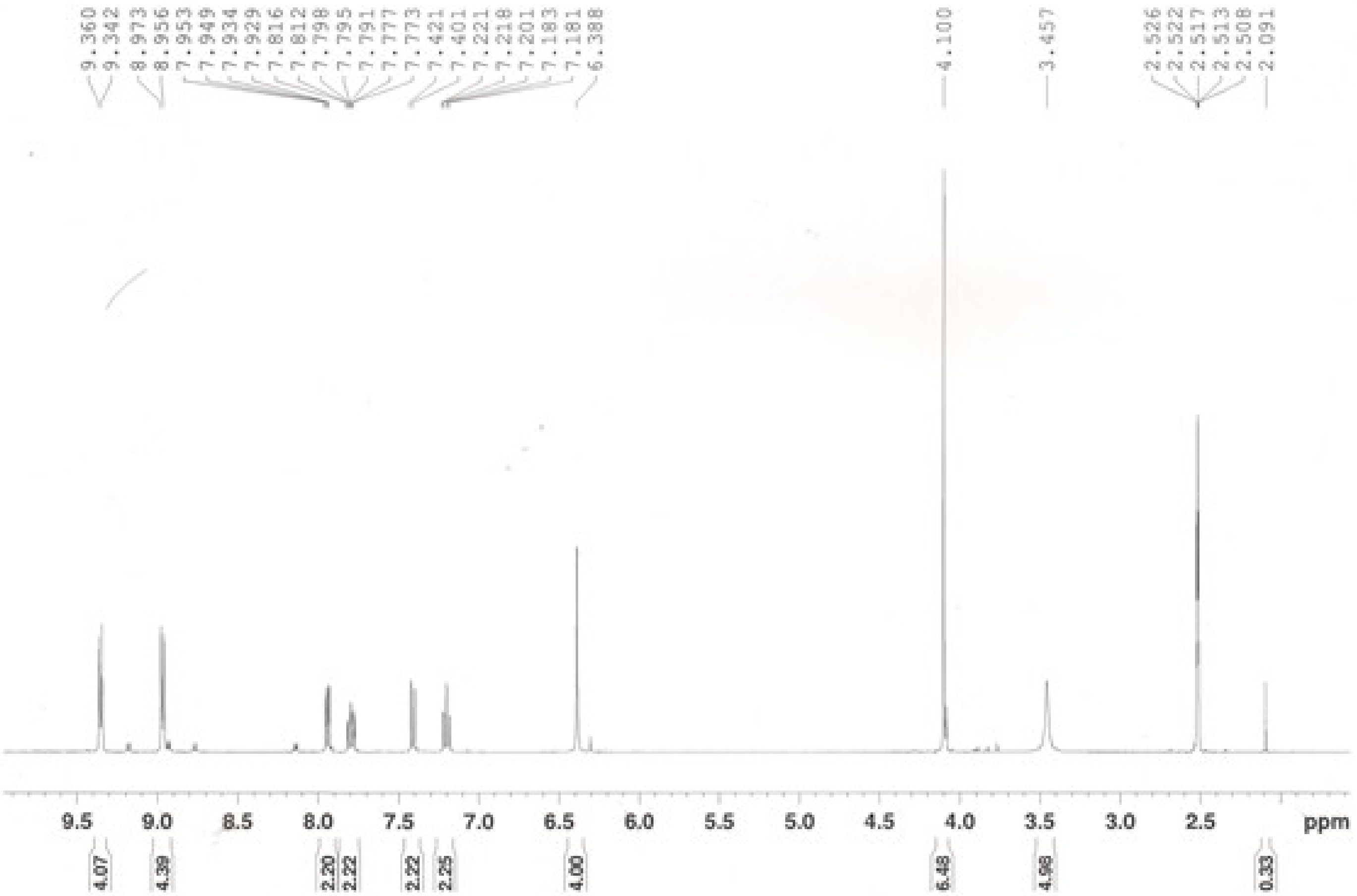

Supplement: Supplementary File 1 [file molecules-19-11572-s001.pdf]
